# Carbon storage of headwater riparian zones in an agricultural landscape

**DOI:** 10.1186/1750-0680-7-4

**Published:** 2012-02-14

**Authors:** Richard D Rheinhardt, Mark M Brinson, Gregory F Meyer, Kevin H Miller

**Affiliations:** 1 Department of Biology, East Carolina University, Mail Stop 551, Greenville, North Carolina, 27858, USA; 2 Coastal Resources Management, East Carolina University, 379 Flanagan, Greenville, North Carolina, 27858, USA; 3 North Carolina Department of Environment and Natural Resources, 1601 Mail Service Center, Raleigh, North Carolina, 27699, USA; 4 Current address: Bureau of Land Management, Grand Staircase-Escalante National Monument, 669 South Highway 89A, Kanab, Utah, 84741, USA

**Keywords:** carbon storage capacity, condition, riparian buffer

## Abstract

**Background:**

In agricultural regions, streamside forests have been reduced in age and extent, or removed entirely to maximize arable cropland. Restoring and reforesting such riparian zones to mature forest, particularly along headwater streams (which constitute 90% of stream network length) would both increase carbon storage and improve water quality. Age and management-related cover/condition classes of headwater stream networks can be used to rapidly inventory carbon storage and sequestration potential if carbon storage capacity of conditions classes and their relative distribution on the landscape are known.

**Results:**

Based on the distribution of riparian zone cover/condition classes in sampled headwater reaches, current and potential carbon storage was extrapolated to the remainder of the North Carolina Coastal Plain stream network. Carbon stored in headwater riparian reaches is only about 40% of its potential capacity, based on 242 MgC/ha stored in sampled mature riparian forest (forest > 50 y old). The carbon deficit along 57,700 km headwater Coastal Plain streams is equivalent to about 25TgC in 30-m-wide riparian buffer zones and 50 TgC in 60-m-wide buffer zones.

**Conclusions:**

Estimating carbon storage in recognizable age-and cover-related condition classes provides a rapid way to better inventory current carbon storage, estimate storage capacity, and calculate the potential for additional storage. In light of the particular importance of buffer zones in headwater reaches in agricultural landscapes in ameliorating nutrient and sediment input to streams, encouraging the restoration of riparian zones to mature forest along headwater reaches worldwide has the potential to not only improve water quality, but also simultaneously reduce atmospheric CO_2_.

## Background

A significant amount of global carbon can be sequestered in forests [1-3] and especially in forest soils [4,5]. This is particularly true for soils in wetlands where decomposition is slower [6,7]. In agricultural regions of the world, many forests along headwater streams, generally first to fourth order (*sensu *[8]), have been completely removed or severely reduced in extent in order to maximize arable cropland. The alterations in buffer zones resulting from forest removal and conversion to agriculture have led to reductions in ecosystem services, including a decline in water quality due to increased soil erosion and a reduced capacity for nutrient uptake and denitrification [9,10], a reduction in habitat quality [11], a reduction in stream particulate organic carbon [12], and an increase in atmospheric CO_2 _[13,14].

It is well known that the aboveground carbon pools of forests gain biomass as they age [15-17] as do forest soils [18,19,5]. However, there is still a paucity of field data for estimating the amount of carbon sequestered as riparian forests age or on how carbon is segregated among various biomass compartments: living vs. detrital, aboveground vs. belowground, or among strata. In order to adequately estimate the potential effects of riparian reforestation on the amount and rate of carbon storage, it would be useful to know to how much carbon is stored in various types of vegetation cover types, in forests of various ages, and in soils as riparian forests develop. Data from headwater riparian forests would be particularly useful because restoration and regeneration of riparian forest not only sequesters atmospheric carbon, but also improves water quality.

## Results and Discussion

The carbon content of riparian zone cover/condition types ranged from 17.9 MgC/ha for Annual Rowcrop agriculture to 241.7 MgC/ha for Mature Forest (> 50 y old) (Table 1) [20,21]. Total carbon content of Mature Forest is 7-13 times that of non-forest condition types (Perennial Herb, Shrub/Sapling, and Annual Rowcrop). The highest proportion of carbon is concentrated in living trees, with 2.6 times as much in Mature Forest than in Regenerating Forest (5-25 y old), while the highest concentration of detrital-based carbon is concentrated in forest soil. These values do not include the carbon content of roots, which were not measured, or carbon content of soil > 10 cm in depth. Roots would be expected to provide 10-15% additional carbon [22] and soil organic carbon to 1 m depth would be at least twice the amount recorded [23].

**Table 1:** Mean stored carbon (MgC/ha) of living and detrital components for condition types in headwater riparian zones

Condition type (n)	Tree	Shrub	Sapling	Herb	Woody Seedling	Vine	Litter	Snag	Large Down Wood	Soil	Total Live	Total Detrital	Total Carbon Stored
Mature Forest (5)	155.7	0.1	0.2	0.1	-	0.2	34.4	8.3	3.6	39.2	156.3	85.4	241.7
Young Forest (5)	59.4	0.0	0.3	0.1	0.1	0.2	20.6	2.4	11.9	33.4	60.1	68.2	128.4
Regenerating Forest (6)	59.2	0.1	0.4	1.3	0.1	0.2	6.8	1.1	4.5	28.6	61.3	41.0	102.3
Recently Clearcut (3)	1.2	0.0	0.1	0.6	0.2	0.1	15.5	4.0	19.3	41.4	2.2	80.3	82.5
Perennial Herb (3)	-	0.2	0.2	3.9	0.4	1.3	0.7	-	-	29.1	6.1	29.8	35.8
Shrub/Sapling (2)	-	-	-	3.6	-	0.0	4.3	-	-	21.0	3.6	25.3	28.9
Annual Rowcrop [21]	-	-	-	0.8	-	-	0.8	-	-	16.3	0.8	17.1	17.9

Mature Forest (> 50 y old), Young Forest (26-50 y), Regenerating Forest (5-25 y), Recently Clearcut (0-5 y). Data derived from [20].

The living and detrital pools of mature Coastal Plain riparian forests are similar in magnitude to other mature temperate forests studied. The 203 MgC/ha for aboveground biomass in Mature Forest is within the range exhibited by a 60-100 y old *Pinus strobus*-dominated forest in Rhode Island, USA (185-301 MgC/ha) [24]. Average annual rate of ecosystem carbon accumulation in the sampled riparian forests was approximately 2.6 MgC/ha y^-1 ^over 80 y, which was a bit higher than other temperate (upland) hardwood forest ecosystems (1.3-2.1 MgC/ha y^-1^)[24], but reasonable considering the riparian forests in this study are seasonally saturated wetlands in a Humid Subtropical climate.

Once carbon values were established for condition types, carbon status was extrapolated over the entire Coastal Plain headwater stream network based on the distribution of condition types along the sampled networks (Table 2) [25,22]. Because the ratio of headwater network length to watershed size in the sampled watersheds was 1.008, the total length of headwater streams in the Coastal Plain was estimated to be about 57,227 km.

**Table 2:** Carbon stored in headwater riparian reaches of sampled riparian zones and extrapolated to the entire Coastal Plain network

Location (*n = *no. of reaches)	Watershed size (km^2^)	Stream network length (km)	MgC/ha (30-m-wide zone)	MgC/ha (60-m-wide zone)	MgC/km stream length (30-m-wide zone)	MgC/km stream length (60-m-wide zone)	Total MgC stored in (30-m-wide zone)	Total MgC stored in (60-m-wide zone)
Cow Swamp (40)	44.5	47.5	94.2	92.3	283	554	13,427	26,299
Crisp Creek (37)	45.9	41.2	84.0	87.0	252	522	10,380	21,514
Lumber (66)	73.7	74.8	104.4	100.5	313	603	23,415	45,070
Current condition of Coastal Plain riparian zones	56,772	57,227	96.3	94.7	289	568	16,529,285	32,512,359
Potential condition of Coastal Plain riparian zones	56,772	57,227	241.7	241.7	0.725	1.450	41,495,029	82,990,058
Difference							24,965,744	50,477,700

Carbon is estimated to be 0.5 times organic matter [25]. Data for Coastal Plain riparian zones based on the weighted average of the three sampled watersheds. Potential carbon storage based on carbon estimated in Mature Forest. Current and potential carbon values of Coastal Plain riparian zones based on extrapolation from sampled networks. Riparian zone widths: 30-m-wide zone includes the 0-15 m bands on both sides of stream; the 60-m-wide zone includes the 0-30 m bands on both sides of stream. "Difference" represents the amount of additional carbon that could potentially be stored in Coastal Plain headwater networks. Total carbon pool would be about 10-15% more than indicated values if large roots (not sampled) were also included [23].

An average of 95 MgC/ha occurred along the 60-m-wide riparian zone of the three sampled Coastal Plain stream networks. This is far less than a potential 242 MgC/ha that would be stored if the entire riparian zone were Mature Forest. Assuming that the three sampled stream networks represent all other rural, headwater, stream networks in Coastal Plain North Carolina, then headwater riparian zones in the Coastal Plain currently store 16.5 to 32.8 TgC, depending on whether the riparian zone is defined as being 30-m wide (16.5 TgC) or 60-m wide (32.5 TgC). These carbon amounts are about 40% of what is possible (Table 2) [25,22] and represent a shortfall of 25.0 to 50.5 TgC (depending on riparian zone width) based on the carbon storage capacity of Mature Forest. This means that if buffer zones of Coastal Plain stream networks are similar, on average, to the condition of sampled networks, then there is potential for storing an additional 25 to 50 TgC (from 30- and 60-m-wide buffers, respectively). Fifty TgC is equivalent to the average annual carbon emissions of about 36,000 homes or 16,000 passenger vehicles (see [26] for other equivalencies). Focusing carbon offsets on riparian zones, particularly in agricultural landscapes, has the advantage of improving water quality of rivers and the estuaries they feed.

Studies that finely compartmentalize living and detrital carbon pools are rare. Even fewer studies provide a chronosequence to depict change in carbon storage among compartments through time. This study is the first to define riparian forest carbon storage by broad cover/condition types related to a forest development sequence and then extrapolate the relative areal extent of those condition types to a larger region to estimate the potential gain in carbon storage that could accrue following full reforestation to mature forest.

## Conclusions

Headwater riparian zones are particularly important hotspots for influencing water quality because 90% of the interface between uplands and aquatic systems occurs in headwater reaches. This makes headwater reaches the major recipient of nonpoint source pollution in agricultural landscapes. However, most headwater reaches in agricultural landscapes are poorly buffered, i.e., either buffers are narrow and/or remnant buffers represent young developmental stages. This situation likely arose from efforts to maximize arable cropland and manage streamside forests as woodlots. Poor riparian buffers have exacerbated water quality problems because the reduction in living and detrital biomass associated with deteriorated buffers has allowed for increased soil erosion and nutrient input in combination with reductions in nutrient uptake and denitrification. By measuring organic matter stored in various condition classes found along streams and randomly sampling headwater reaches to identify the relative distribution of those condition classes along streams, this study found that riparian buffer zones of headwater streams in Coastal Plain North Carolina store only 40% of the carbon they could potentially store if mature forest buffered all streams. Many headwater streams in the agricultural landscapes worldwide are also poorly buffered. Encouraging the development of older-aged forests, particularly mature forest, in headwater reaches of agricultural regions has the potential to improve ecosystem services like water quality and habitat quality, while simultaneously sequestering a huge amount of atmospheric carbon. Thus, restoration of riparian forest buffer zones in agricultural landscapes, particularly in headwater reaches, should become a priority worldwide.

## Methods

**Figure 1:**
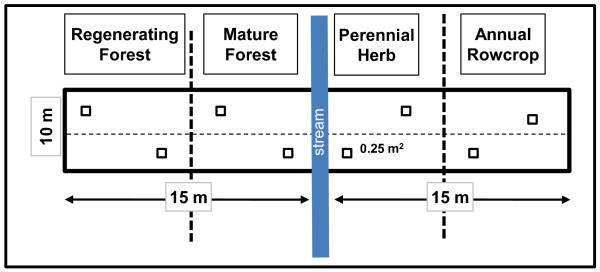
**Layout of 10 × 30 m belt transects for sampling biomass of riparian condition types**. Locations of imbedded 0.25 m quadrats are small squares (n = 6) within belt transects. Diagram illustrates a hypothetical juxtaposition of four condition types.

A space-for-time approach was used to obtain data on storage of carbon as riparian forests develop from agricultural fields to shrub/saplings to mature forest. Riparian ecosystems in Coastal Plain North Carolina are naturally vegetated by forest, and so mature forest is the endpoint in vegetation development. However, riparian zones along most headwater portions of stream networks have been converted to other uses (mostly agriculture) and are thus in varying stages of development. After extensive surveys of headwater riparian zones, it was found that riparian vegetation could be partitioned into one of eight easily-recognizable age and cover-related condition types, which correspond to a chronosequence from agriculture to mature forest: (1) Annual Rowcrop (corn, soybean, cotton), (2) Perennial Herb (usually fallow field of grasses and perennial forbs), (3) Shrub/Sapling, (4) Recently Clearcut Forest (< 5 y since last clearcut), (5) Regenerating Forest (5-25 y old), (6) Young Forest (26-50 y old), and (7) Mature Forest (> 50 y old). Biomass was determined for these condition types, by biomass category (roots excluded), in 30-m long by 10-m wide belt transects along 18 stream reaches in agricultural, Coastal Plain North Carolina. Each sampled reach consisted of one to several condition classes (Figure 1). Detailed methods for determining biomass by condition type, further partitioned by living and detrital biomass categories, are explained in companion studies [20,27]. Assuming that dry plant biomass and soil organic matter is about 50% organic carbon [28,25], biomass was converted to carbon content for each of the sampled compartments.

After carbon was quantified for each condition class, 100-m long reaches (n = 143) were randomly chosen in three rural Coastal Plain stream networks, all embedded in agricultural drainage basins, ranging in size from 44.5 to 73.7 km^2^. Sampled reaches constituted about 10% of stream network length [29]. However, before defining the stream network, it was necessary to ensure that all intermittent streams in the network were included. This was a challenge because most headward, first order streams are often not even depicted as streams on United States Geologic Survey (USGS) 1:24 K topographic maps and associated digital files, even some that flow year-round during wet years. Considering that many headwater streams are missing on USGS maps, additional headward portions of intermittent, groundwater-driven reaches in the three sampled stream networks were manually mapped, using hydrogeomorphic criteria. Detailed methods of this mapping exercise are provided in [29].

After mapping condition classes along a 100-m reach, the cover of condition classes were estimated for each side of the stream separately with respect to two riparian zone bands, extending perpendicular from the stream edge: a 0-15 m band and a 15-30 m band on each side [20]. Sometimes more than one condition type occurred within one of the two riparian bands. When this occurred, the types were apportioned to the band in approximate proportion to their relative cover. Condition-type data for each 100-m reach within a band were then converted to total stored carbon. Carbon storage data from both sides of the stream reach were combined: inner bands combined to provide carbon storage data for a 30-m-wide zone, and all data combined to obtain data for a 60-m-wide zone. Since the reaches were 100 m long, the data for the two riparian zones were for 1/3 ha (for the 30-m-wide zone) and 2/3 ha (for the 60-m-wide zone).

Assuming that the three sampled headwater networks represented the condition of all Coastal Plain riparian zones, the carbon content of the sampled riparian zones was extrapolated to the entire North Carolina Coastal Plain headwater network. Potential carbon content was then estimated by calculating the amount of carbon that could be stored if all riparian zones were allowed to regenerate to mature forest. Thus, the difference between potential and current carbon content of riparian zones represents the amount of additional carbon that could potentially be stored in Coastal Plain riparian zones.

The main assumption of this approach is that the sampled stream networks represent the condition of the entire Coastal Plain headwater network. Even though the sampled watersheds had been identified *a priori *by the state of North Carolina as being watersheds with impaired water quality (*sensu *Clean Water Act, Section 303d), based on the authors' considerable field experience in the Coastal Plain, it did not appear that riparian zone condition in the sampled stream networks differed substantially from other headwater stream networks.

The main limitation of this study is that the sampled watersheds represent only the rural (agricultural) and headwater portions of the Coastal Plain, i.e., urban portions of networks and portions of networks larger than 4^th ^order were not included in this analysis. However, the headwater reaches of mapped 1^st^-4^th ^order steams represent > 90% of all stream length in Coastal Plain North Carolina (Rheinhardt, unpublished data), most higher order stream reaches (> 4^th ^order) are adequately buffered by forest (although not everywhere by mature forest), and the proportion of urban stream length is rather small in relation to rural stream length.

Hydrologic conditions are known to affect rates of carbon sequestration. Net primary production has been found to significantly differ between wet sites (permanently to semi-permanently saturated) and drier sites (seasonally saturated to upland sites), but not between seasonally saturated and upland sites [30]. Hydrologic conditions of the riparian zones of this study ranged from seasonally saturated to upland and so no differences were expected in sequestration rates across the moisture gradient, i.e., carbon storage in similarly-aged forest stands would not be expected to significantly differ between the wetland portion of the riparian zone and the upland portion of the zone. Further, any differences in carbon sequestration that could be attributable to soil moisture differences would be incorporated in the species-specific allometric and regression equations that were used to estimate biomass, since each species tends to be concentrated along certain portions of the moisture gradient from wetland to upland.

## List of abbreviations

USEPA: United States Environmental Protection Agency; NCAS: North Carolina Agricultural Statistics

## Competing interests

The authors declare that they have no competing interests.

## Authors' contributions

RR helped conceive the study, participated in study design, fieldwork and data analysis, and drafted the manuscript. MB helped conceive the study, participated in study design, and fieldwork. GM participated in field work, lab work, data entry and analysis, and GIS work. KM: participated fieldwork, lab work, data entry and analysis, and GIS work. All authors read and approved the final manuscript.
